# Investigation of polycapillary half lenses for quantitative confocal micro-X-ray fluorescence analysis

**DOI:** 10.1107/S1600577522009699

**Published:** 2022-10-21

**Authors:** Michael Iro, Dieter Ingerle, Martin Radtke, Ana Guilherme Buzanich, Peter Kregsamer, Christina Streli

**Affiliations:** a Atominstitut, TU Wien, Stadionallee 2, Vienna 1020, Austria; b Bundesanstalt für Materialforschung und -prüfung (BAM), Richard-Willstätter-Straße 11, 12489 Berlin, Germany; Tohoku University, Japan

**Keywords:** polycapillary optics, confocal micro-XRF, X-ray fluorescence, 3D elemental analysis, X-ray optics

## Abstract

The use of polycapillary optics in confocal micro-X-ray fluorescence analysis (CMXRF) enables the destruction-free 3D investigation of the elemental composition of samples. The energy-dependent transmission properties, concerning intensity and spatial beam propagation of three polycapillary half lenses, which are vital for the quantitative interpretation of such CMXRF measurements, are investigated in a monochromatic confocal laboratory setup at the Atominstitut of TU Wien, and a synchrotron setup on the BAMline beamline at the BESSY II Synchrotron, Helmholtz-Zentrum-Berlin. The empirically established results, concerning the intensity of the transmitted beam, are compared with theoretical values calculated with the *polycap* software package and a newly presented analytical model for the transmission function. The resulting form of the newly modelled energy-dependent transmission function is shown to be in good agreement with Monte Carlo simulated results for the complete energy regime, as well as the empirically established results for the energy regime between 6 keV and 20 keV. An analysis of possible fabrication errors was conducted via pinhole scans showing only minor fabrication errors in two of the investigated polycapillary optics. The energy-dependent focal spot size of the primary polycapillary was investigated in the laboratory via the channel-wise evaluation of knife-edge scans. Experimental results are compared with data given by the manufacturer as well as geometric estimations for the minimal focal spot size. Again, the resulting measurement points show a trend in agreement with geometrically estimated results and manufacturer data.

## Abstract 2

A new analytical model for the transmission function of polycapillary half-lens optics is presented. The model is compared with measurement results taken at the BAMline beamline at BESSY II and at TU Wien, as well as Monte Carlo simulations.

## 1. Introduction

Since their development by Kumakhov (1990 ▸), polycapillary optics have been widely used in X-ray fluorescence analysis (XRF) to focus or parallelize X-ray beams (Kumakhov, 2000 ▸; MacDonald, 2010 ▸; Matsuda *et al.*, 2008 ▸). With the use of two polycapillary optics – one between the X-ray source and the sample to focus the primary beam, and the other between sample and detector to limit the visible area of the detector – a confocal setup can be realized (Nakazawa & Tsuji, 2013*a* ▸; Ingerle *et al.*, 2020 ▸; Haschke, 2014 ▸), allowing for 3D imaging of samples from various fields of study [*e.g.* biology, geology, cultural heritage *etc.* (Fittschen & Falkenberg, 2011 ▸; Šmit *et al.*, 2004 ▸; Nakano *et al.*, 2011 ▸; Nakazawa & Tsuji, 2013*b* ▸; Sun *et al.*, 2010 ▸, 2020 ▸; Kanngießer *et al.*, 2005 ▸)]. Quantitative interpretations of such measurements rely heavily on the transmission properties of the polycapillary optics used (Mantouvalou *et al.*, 2012 ▸). Information about these properties from the manufacturer is often insufficient for an exact quantitative evaluation of confocal micro-X-ray fluorescence analysis (CMXRF) measurements.

In this paper, as a step towards 3D elemental quantification with a CMXRF setup, the transmission properties of three polycapillary half lenses (PC-50, PC-236, PC-246) were established, with experiments carried out in the laboratory and at a synchrotron source. Two of the investigated lenses (PC-236, PC-246) are used in the confocal laboratory setup reported by Ingerle *et al.* (2020 ▸). The results are compared with data given by the manufacturer and simulated values established with the software *polycap* (Tack *et al.*, 2020 ▸).

In addition to previously reported similar investigations of polycapillary lenses (Matsuda *et al.*, 2008 ▸; Wolff *et al.*, 2009 ▸; Haschke & Haller, 2003 ▸), we present newly developed analytical models for the estimation of the global divergence and the transmission ratio of the polycapillary optics.

## 2. Experimental

The properties of the spot size close to the focal distance of the polycapillary were investigated using knife-edge scans in a monochromatic laboratory setup. For a full investigation of the spot size further outside the focal distance, the transmission ratio and possible small fabrication defects, further experiments were conducted in a setup at the BAMline beamline at the BESSY II Synchrotron of the Helmholtz-Zentrum-Berlin (HZB) (Riesemeier *et al.*, 2005 ▸).

Two polycapillary optics by Fischer GmbH (Sindelfingen, Germany) and one by XOS (New York, USA) were investigated. The data given by the manufacturers are shown in Table 1 ▸, where *f* is the focal distance, *L* is the length of the optic, and *D*_in_ and *D*_out_ are the input and output diameters of the polycapillary in the focusing direction, respectively.

### 2.1. Laboratory setup

**Figure 1:**
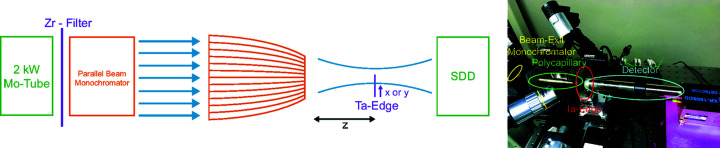
Experimental laboratory setup at the Atominstitut of TU Wien.

The setup used for the knife-edge scans is an adapted version of the setup presented by Ingerle *et al.* (2020 ▸). It uses a 2 kW water-cooled X-ray-diffraction glass tube with an Mo anode and a 1D parallel beam multilayer X-ray optic produced by AXO (Dresden, Germany) for Mo *K*α, to monochromatize and collimate the beam before it enters the polycapillary. The detector was placed in the line of the focused beam. Between the polycapillary optic and detector a Ta-edge was moved in different directions in and out of the beam. A sketch of the setup is given in Fig. 1 ▸. The results of these measurements are presented in Section 3.1 of the *Results and Discussion* section.

### 2.2. Synchrotron setup

**Figure 2:**
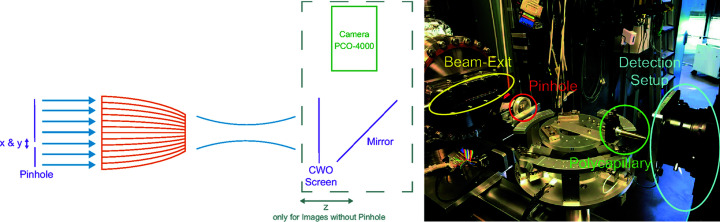
Experimental setup on the BAMline beamline at the BESSY II synchrotron, at the HZB.

The experiments using a synchrotron source were conducted on the BAMline beamline at the BESSY II synchrotron, at the HZB. The synchrotron was operated in single-bunch mode. To monochromatize the beam, a double-multilayer with fixed-exit setup was used. The beam properties were measured with a pco.4000 camera, filming a cadmium tungstate screen (CWO screen) at which the beam was directed. For the investigation of beam properties, far-from-focal-distance images of the beam were taken at different distances of approximately 1–2 cm between the polycapillary exit window and the camera (see *z* in Fig. 2 ▸, left). Possible small fabrication defects were investigated with pinhole scans, exposing only a small area (pinhole diameter 250 µm) of the polycapillary optic to the beam. The results of these measurements are presented in Sections 3.2–3.4 of the *Results and Discussion* section.

## 3. Results and discussion

### 3.1. Near-focal-distance investigation in the laboratory

**Figure 3:**
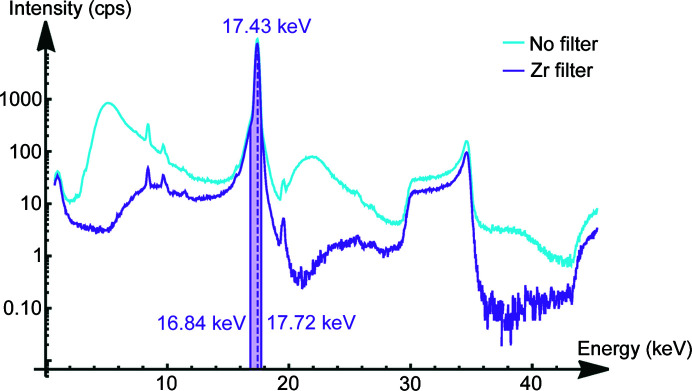
Beam spectrum in logarithmic scale with and without a Zr filter. The marked ROI holds approximately 87% of the total beam intensity. The X-ray tube was operated at 50 kV and 5 mA.

The energy spectrum of the beam, transmitted by the primary polycapillary (PC-246), was measured using the laboratory setup (see Fig. 1 ▸). With the use of an ∼25 µm Zr-filter, in addition to the parallel beam multilayer optic, a very narrow spectral bandwidth is achieved that justifies the assumption of a monochromatic beam (approximately 87% of the total intensity is in a narrow 1 keV band around Mo *K*α, see Fig. 3 ▸).

Although the only significant signal is in the region of interest (ROI) around Mo *K*α, direct measurement of the beam provides enough signal for a channel-wise evaluation of knife-edge scans using a Ta-edge of approximately 1 mm thickness.

Under the assumption of a beam profile similar to a multivariate normal distribution (see Section 3.2), the focal spot size can be approximated by the full width at half-maximum (FWHM) of the respective probability density function (PDF) for the measured cumulative density function (CDF) of a normal distribution (Haschke, 2014 ▸).

**Figure 4:**
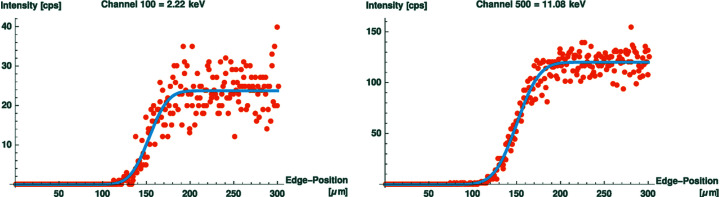
Knife-edge scans for different channels (horizontal). As shown for channel 100 on the left, channels in the low- and high-energy range do not give a statistically relevant signal suitable for evaluation. Channels between 6.2 keV and 18.0 keV, such as channel 500 shown as an example on the right, are suitable for a channel-wise evaluation.

Fig. 4 ▸ shows the measured count rates (orange) for two separate single channels and the fitted cumulative density function for a Gaussian distribution (blue). The count rate in channel 500 (right) is sufficient for a channel-wise evaluation, whereas channels in the low-energy regime, such as channel 100 (left), do not measure a statistically relevant signal, making the result of the fit arbitrary to some degree. Sensible results in reasonable measurement times can be found for energies between 6.2 keV and 18.0 keV. To avoid signals overstressing the detector, the X-ray tube was operated with a low power at 50 kV and 5 mA and attenuated with an ∼25 µm Zr filter (see Fig. 3 ▸).

**Figure 5:**
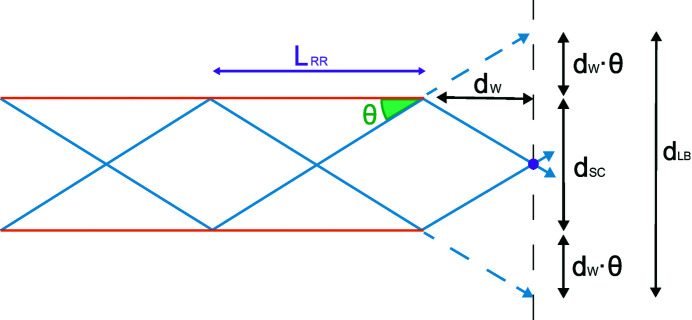
Multiple reflections and estimates of a lower boundary for the spot size close to the focal distance of a straight monocapillary optic.

Neglecting misorientation of different single capillaries, a lower boundary for the polycapillary spot size *d*_LB_ can be estimated using the parameters of a single capillary,

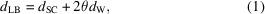

where *d*_SC_ is the diameter of a single capillary, θ is the angle of reflection for the dominant part of the radiation of a given energy *E* and *d*_W_ is the working distance of the polycapillary optic (see Fig. 5 ▸).

**Figure 6:**
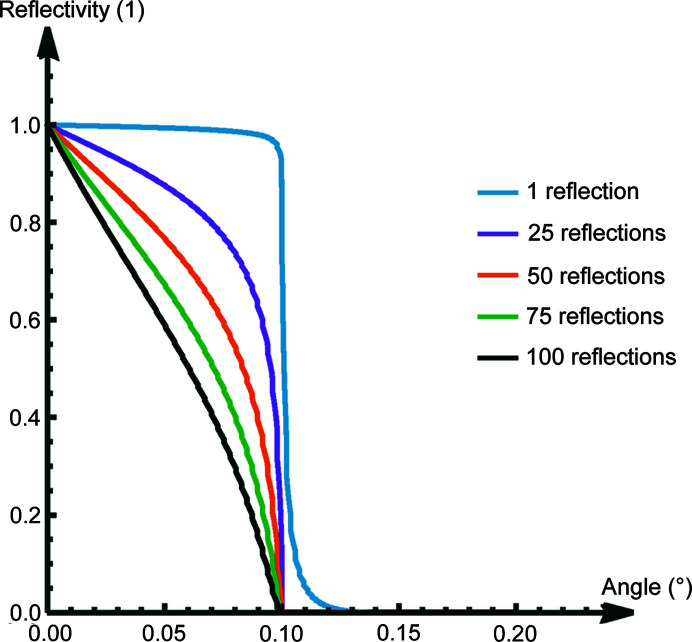
Calculated total reflectivity for a reflection on an SiO_2_ surface for multiple reflections of X-rays with an energy of 17.4 keV.

The large average number of reflections in the optic – in our case approximately 40 – must be considered for the choice of θ. The total reflectivity for multiple reflections being much smaller than 1, as opposed to approximately 1 for a single reflection, results in the dominant amount of transmitted radiation that passes the optic being reflected with an angle considerably smaller than the critical angle. Fig. 6 ▸ shows the calculated angle dependence reflectivity of a thick plane SiO_2_ mirror surface taken from https://henke.lbl.gov/optical_constants/mirror2.html as well as the calculated values for multiple reflections.

**Figure 7:**
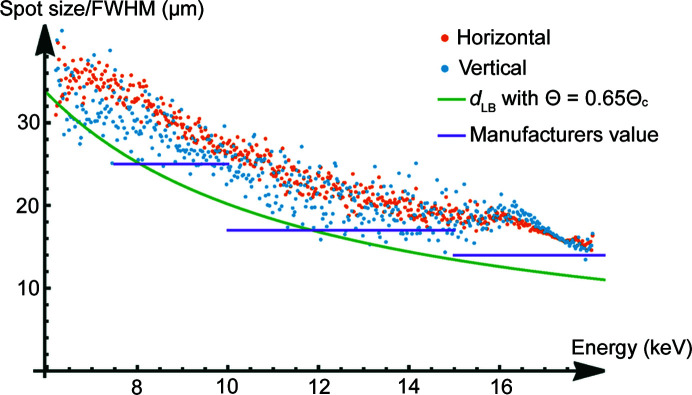
Energy-dependent spot size of PC-246 in the focal distance for horizontal and vertical knife-edge scans.

Fig. 7 ▸ shows the measured spot sizes for different energies at the focal distance of the polycapillary optic PC-246 (5.1 mm) compared with values given by the manufacturer and a lower boundary estimation for the spot size considering multiple reflections.

The measurement results in the horizontal and vertical directions of the knife-edge are in good agreement, as shown in Fig. 7 ▸. Small variations are to be expected due to the uncertain orientation of the hexagonal structure of the polycapillary.

### 3.2. Far-from-focal-distance investigation at the synchrotron

**Figure 8:**
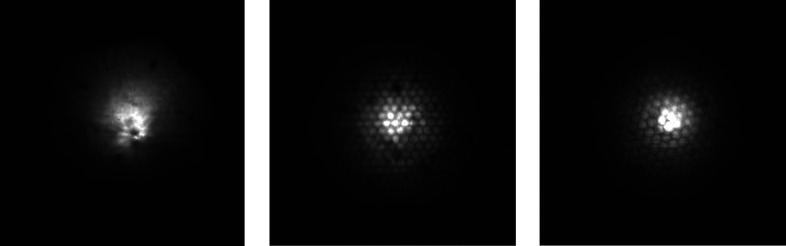
Beam intensity distribution for a primary beam energy of 12 keV for three different polycapillary optics PC-50 (left), PC-236 (middle) and PC-246 (right).

Images of the beam were taken for different distances (approximately 1–2 cm) between the CWO screen and the polycapillary optic at different energies for three different polycapillary optics (see Figs. 8 ▸, 9 ▸ and 10 ▸).

**Figure 11:**
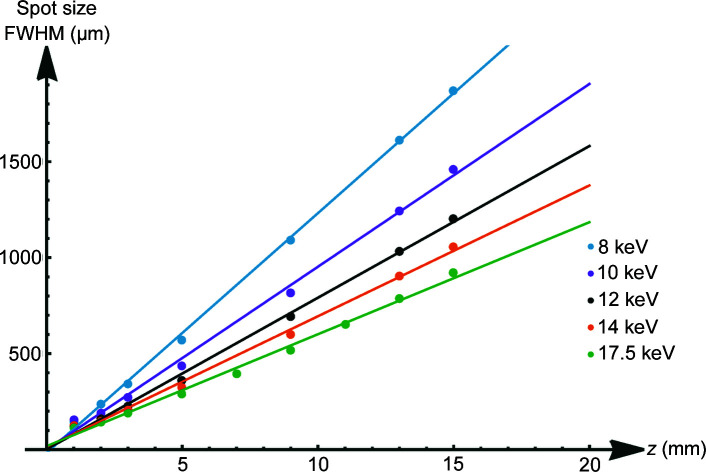
Divergence far from the focal distance of PC-246 for different energies.

**Figure 12:**
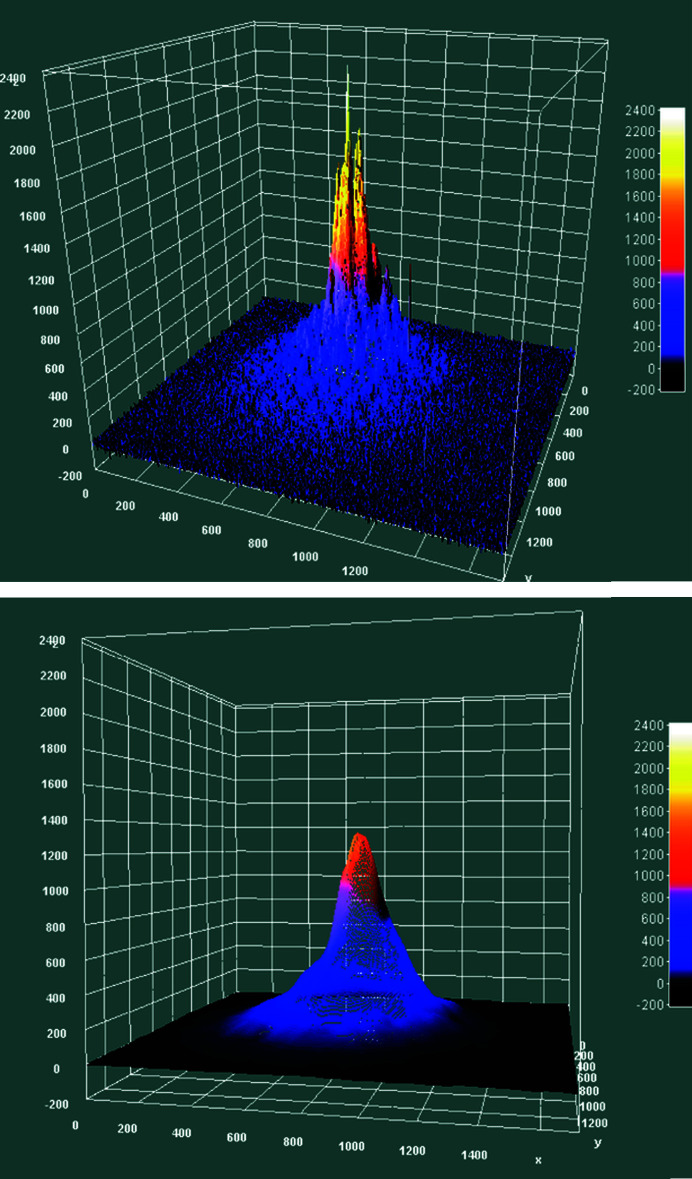
3D beam intensity distribution for PC-246 for the 12 keV primary beam unsmoothed (top) and smoothed (bottom).

All images show the hexagonal bunch structure of the polycapillary optics very well. As can be seen in the images, the use of the screen and camera available at the synchrotron allows for a much more detailed investigation of the 2D intensity distribution of the beam than knife-edge scans, considering only two axes and neglecting the orientation of the capillary with respect to rotation around the beam axis. As expected, a smaller spot size for higher energies is observed (see Fig. 8 ▸). The energy dependence of the out-of-focus spot size is shown in Fig. 11 ▸. In order to better establish the form of the beam intensity distribution, a 3D model of the beam was created with *ImageJ* (Schindelin *et al.*, 2012 ▸) (see Fig. 12 ▸).

**Figure 13:**
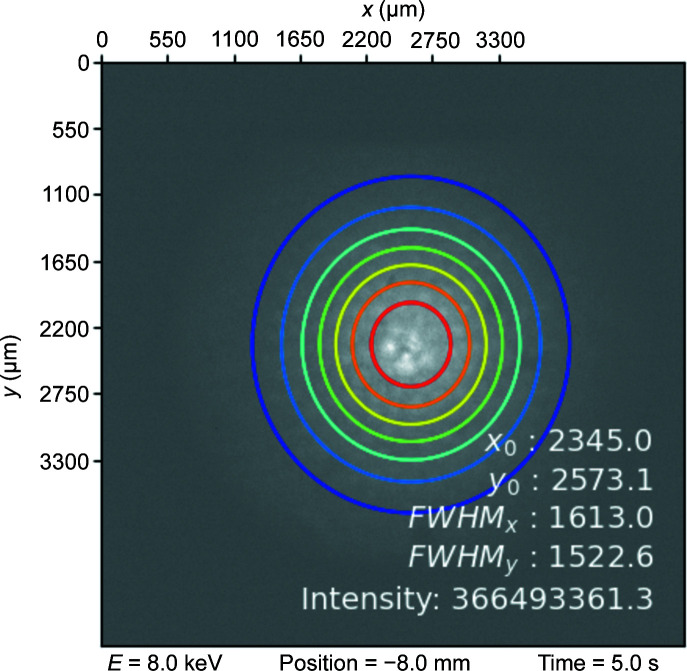
Fitted multivariate normal distribution parameters.

While the intensity distribution of the beam is not strictly a multivariate normal intensity distribution (see Fig. 12 ▸, left), smoothing the measured data with the *Interactive 3D Surface Plot function* of *ImageJ* shows a near multivariate normal distribution (see Fig. 12 ▸, right). Thus, centre and standard deviation of a 2D normal distribution were established by an algorithm calculating the moments of the data and optimizing the established initial guesses for the parameters of the distribution (SciPy, 2022 ▸; Virtanen *et al.*, 2020 ▸). Such a fit is shown in Fig. 13 ▸.

**Figure 14:**
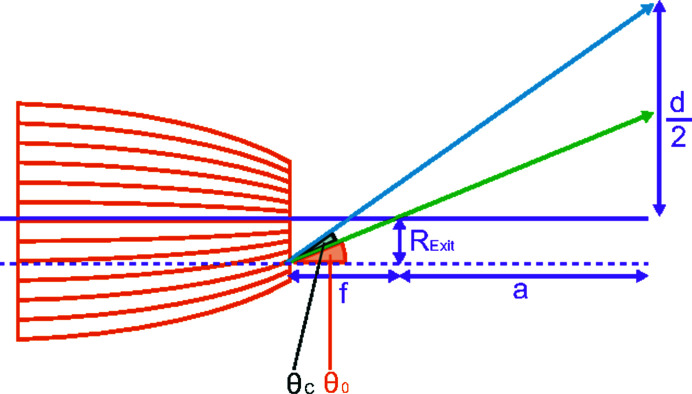
Sketch of the divergence far from the focal distance.

The calculated parameters can be used to determine the global divergence of the polycapillary optic, *i.e.* the beam shape far outside the focal distance, which is additionally dependent on the curvature of the optic [see *d* in Fig. 14 ▸ and equations (2) and (3)] compared with the local divergence close to the focal distance predominantly dependent on the critical angle and therefore the beam energy [see *d*_LB_ in Fig. 5 ▸ and equation (1) (MacDonald, 2010 ▸)].

The energy-dependent divergence far outside the focal distance *d*(*E*), measured as the FWHM of the spot, can be written as (see Fig. 14 ▸)

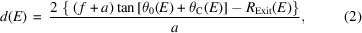

where *f* is the focal distance of the optic, *a* is the distance of the measured spot to the focal point, θ_0_(*E*) is the angle between the tangent to the curvature radius at the polycapillary exit and the optical axis, θ_C_(*E*) is the critical angle, and *R*_Exit_(*E*) is the distance between the optical axis and the ‘outermost’ capillary with a relevant transmittance for radiation of a given energy *E*.

For large *a* and small θ_0_(*E*) and θ_C_(*E*), we can write 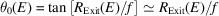 . We get

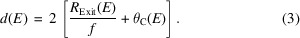

**Figure 15:**
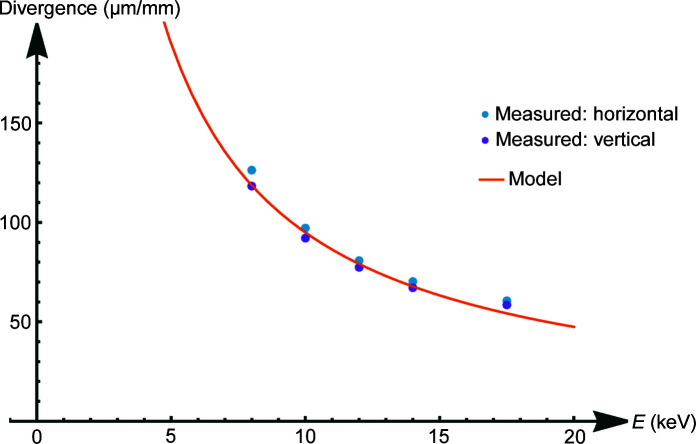
Energy dependence of the divergence far from the focal distance.

Considering the lower number of outer capillaries, *R*_Exit_(*E*) turns out to be proportional to the inverse energy *E* (see Fig. 15 ▸),

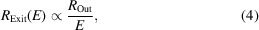

with *R*_Out_ being the polycapillary exit radius. Fig. 15 ▸ shows a comparison of this model with the measurement results.

### 3.3. Transmission

**Figure 16:**
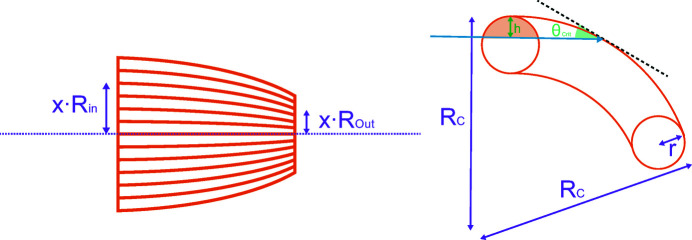
Parametrization of the polycapillary optic (left) and sketch of the bending radius of a single capillary (right).

The shape of the transmission function is dominantly defined by two effects. In the low-energy regime, the average number of reflections of an X-ray inside the capillary – in our case approximately 40 – reduces the number of transmitted photons (see Fig. 6 ▸). In the high-energy regime, the number of transmitted rays is reduced due to a higher number of rays hitting the surface in the outer capillaries under an angle larger than the critical angle (see Fig. 16 ▸) (Haschke, 2014 ▸).

These effects have been qualitatively stated previously and the total transmission function is usually optically classified as a Gumbel function (Haschke, 2014 ▸). Alternatively we present, to our knowledge, the first in-depth analytical model for an estimate of the transmission function, not based on the high computational effort of tracing single rays through the optic (Tack *et al.*, 2020 ▸; Chi, 2020 ▸). Assuming the total transmission function as a product of both mentioned effects one can write

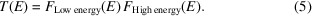

The high-energy part can be derived by calculating the ratio of the illuminated area, which fulfils the condition that the first reflection is at or below the critical angle (see Fig. 16 ▸, right) and the total illuminated area.

Using formulae for circular segments, the bending radius of a single capillary *R*_C_ can be written as

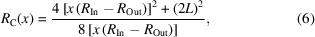

where *R*_In_ and *R*_Out_ are the polycapillary entrance and the exit radii, *L* is the length of the optic, and *x* is the ratio of the distance of the single capillary to the optical axis and the radius. The height *h* and the area *A* of the illuminated area which fulfils the critical condition can be written as

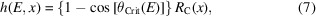




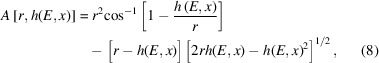

where θ_Crit_ is the critical angle, *E* is the energy of the radiation and *r* is the entrance radius of a single capillary. Considering that the number of single capillaries increases with the distance to the optical axes *x* (note that the area of a circle with radius *r* can be found by the integral), the high-energy part of the transmission function for the complete polycapillary is then given by

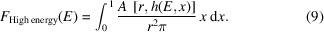

The low-energy part can be written as

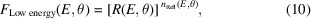

where *R*(*E*, θ) is the reflectivity of an X-ray photon with the energy *E* hitting a surface of SiO_2_ under the angle θ. A detailed explanation of the calculation of *R*(*E*, θ) has been given by Klockenkämper & von Bohlen (2015 ▸). An approximation for the number of reflections *n*_Refl_ can be found by calculating the reflections in a straight monocapillary,

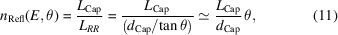

where *L*_Cap_ is the capillary length, which can be approximated by the length of the polycapillary optic, *L*_RR_ is the length between two reflections (see Fig. 5 ▸), *d*_Cap_ is the entry diameter of a single capillary and θ is the angle of reflection. An approximation of the expected value for the angle can be found by considering the reflectivity *R*(*E*, θ) as a weight function for the angle,

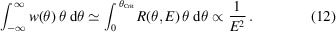

**Figure 17:**
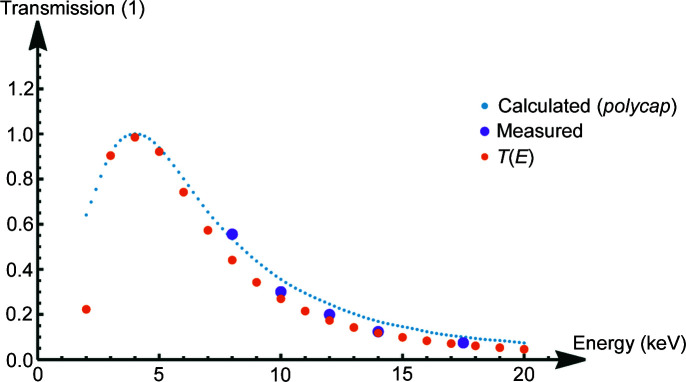
Model for the transmission function compared with single energy measurements made at the synchrotron and Monte Carlo simulations performed with *polycap*. The calculated and simulated values of the various energies are normalized to the maximum transmission at approximately 4 keV. The measured values are scaled accordingly to illustrate the trend of energy dependence.

Therefore, one can assume an expected angle of reflection θ = θ_Crit_/*E* ^2^ in equation (10). With this the total transmission function *T*(*E*) shows its typical shape (see Fig. 17 ▸).

Results for the transmission were established using the 2D Gauss fits of the measurements at the synchrotron (see Fig. 13 ▸). Such measurements were conducted for different energies between 6 keV and 20 keV. The respective intensities (*i.e.* the volume under the established multivariate normal distribution) were normalized to pinhole measurements for each energy (*i.e.* the volume under the established cylinder, using a pinhole with 250 µm diameter). This results in relative values for the transmission.

These values (red) are compared with simulated results using the *polycap* code (blue) and the analytical model presented in this paper (orange). The measured values are scaled accordingly to illustrate the trend of the energy dependence.

The measured values for the transmission are in good agreement with values taken from a Monte Carlo simulation with *polycap* code, as well as values generated with the analytical model presented.

### 3.4. Fabrication defects

**Figure 18:**
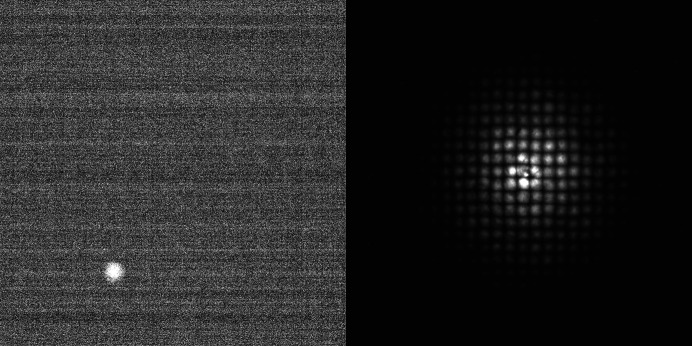
Pinhole scans for PC-246 at a beam energy of 8 keV. Image of a single pinhole position (left). Combined image of different pinhole positions (right).

To better establish small fabrication defects (*i.e.* reduce the smearing of the beam from different parts of the polycapillary), images for different positions of a pinhole, with a pinhole diameter 250 µm, in front of the polycapillary optic, were combined, using the maximal value of each pixel in order to create a full picture of the beam (see Fig. 18 ▸).

This shows possible fabrication defects. To exclude image artefacts stemming from the primary beam, only artefacts visible in the images for all energies are considered capillary artefacts.

**Figure 19:**
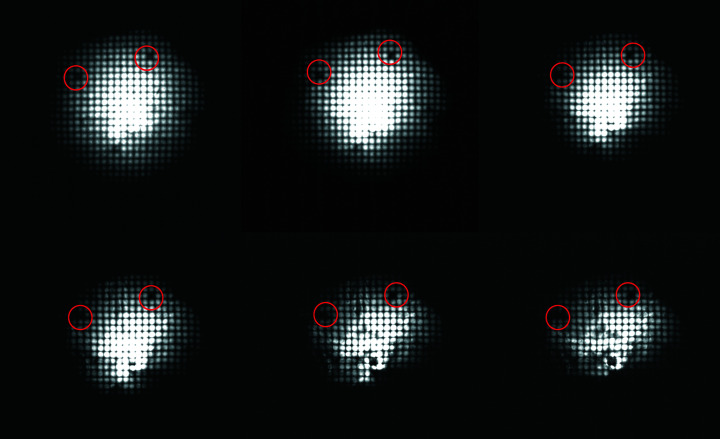
Small fabrication defects of PC-50 identified at beam energies (top-left to bottom-right) of 8 keV, 10 keV,12 keV, 14 keV, 17.5 keV, 20 keV.

In PC-50 and PC-236, small fabrication defects could be identified (see Fig. 19 ▸). The overall form of the transmitted beam in Fig. 19 ▸ is slightly mangled due to a small tilt of the capillary optic which is represented stronger for higher energies.

PC-246 did not show any visible fabrication defects. The overall good fabrication quality of all the optics, verified by the pinhole scans, justifies the assumption of a multivariate intensity distribution of the beam.

## 4. Conclusions

Three different polycapillary optics were characterized experimentally. The measured data for the energy-dependent spot size in the focal distance, established via the channel-wise evaluation of knife-edge scans, conducted in the laboratory setup, show a good agreement regarding the 1/*E* trend of the focal spot size, with data given by the manufacturer as well as geometrical estimations for the minimal focal spot size for a given energy. While this generally good agreement can be seen as the comparable trend of the empirical and estimated values in Fig. 7 ▸, it is also clearly visible that the absolute deviations for single energies from the manufacturer data and the geometric estimations make an exact investigation necessary for quantitative CMXRF measurements. To illustrate this Table 2 ▸ shows the values for three different energies read from Fig. 7 ▸.

Although the trend of all curves shows a similar behaviour and deviations of up to 20% between manufacturer values and can generally be considered small, a wrong estimation can have a significant influence on quantitative interpretations of CMXRF measurements. Furthermore, the deviations can be even larger for energies on the border of the energy interval given by the manufacturer; for example, a spot size of 25 µm for 7.5 keV may be given compared with approximately 30 µm (vertically) × 35 µm (horizontally) leading to a mis-estimation of the spot size of up to 30%.

Spot sizes in distances much larger than the focal distance were measured using the synchrotron setup and compared with Monte Carlo simulated data using the *polycap* code.

Additionally, a newly developed analytical model for the energy-dependent transmission function and global divergence was presented and verified by comparison with the measured and Monte Carlo simulated results. Again, all measured, calculated and simulated results show the same energy trend (see Fig. 17 ▸). All transmission results are relative to each other, leaving a linear scaling factor for the curves.

This characterization of the capillary optics is an important step towards the quantitative interpretation of measurements with the monochromatic CMXRF setup, presented by Ingerle *et al.* (2020 ▸), applicable in the laboratory and at the synchrotron.

The good agreement of the measured data and values calculated with the *polycap* code supports the applicability of this software for the development of a new voxel-based Monte Carlo ray-tracing code for the quantitative interpretation of CMXRF, which will be presented in a future publication.

## Figures and Tables

**Figure 9:**
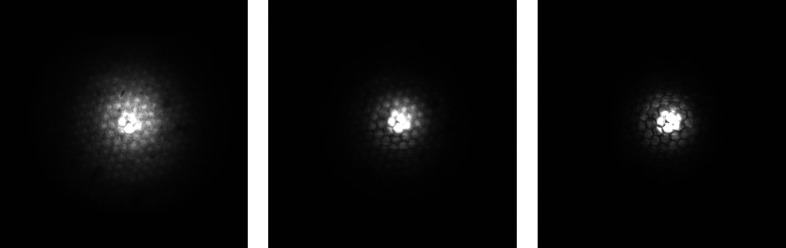
Beam intensity distribution for PC-246 for the primary beam energies 8 keV (left), 12 keV (middle) and 17.5 keV (right).

**Figure 10:**
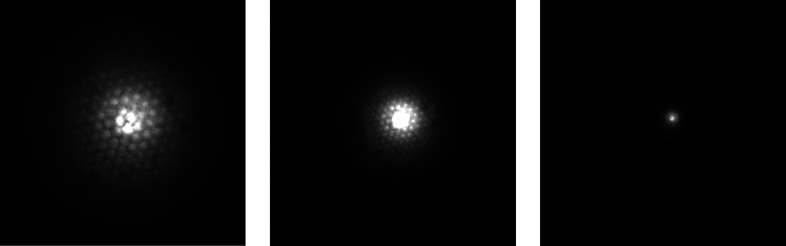
Beam intensity distribution for PC-246 for the primary beam energy 10 keV for different distances (see *z* in Fig. 2 ▸). With 6 mm distance between the left and middle images and 6 mm distance between the middle and right images.

**Table 1:** Properties of the investigated polycapillary optics as given by the manufacturers

	PC-246	PC-236	PC-50
Manufacturer	Fischer	Fischer	XOS
Serial number	246mls26	236mls13	5632
*f* (mm)	5.1	4.9	5.0
*L* (mm)	39.4	40.3	<50.0
*D*_in_ (mm)	7.4	6.35	7.4
*D*_out_ (mm)	2.15	1.9	
Spot size 7.5–10 keV (µm)	25	25	
Spot size 10–15 keV (µm)	17	20	
Spot size 15–20 keV (µm)	14	15	
Spot size 20–25 keV (µm)	14	15	

**Table 2:** Comparison of spot sizes measured, given by the manufacturer and geometrically estimated

	Spot size (µm)		
	8.75 keV	12.5 keV	17.5 keV
Measured values	30	21	16
Manufacturer data	25	17	14
Estimated lower boundary	23	16	11.5
